# The complete mitochondrial genome of *Amesia sanguiflua* (Lepidoptera, Zygaenidae)

**DOI:** 10.1080/23802359.2020.1720535

**Published:** 2020-02-03

**Authors:** Xiaoyu Zhang, Ling Tang, Juan Chen, Ping You

**Affiliations:** College of Life Science, Shaanxi Normal University, Xi’an, Shaanxi, P. R. China

**Keywords:** *Amesia sanguiflua*, mitochondrial genomes, Zygaenidae, Zygaenoidea, phylogenetic analysis

## Abstract

*Amesia sanguiflua* (Lepidoptera, Zygaenidae) is found in northern India, Myanmar, Indochina, Malay Peninsula, Sumatra, Java, and China. In the present study, we sequenced the complete mitochondrial genome of *A. sanguiflua*. The mitochondrial genome was 15,203 bp in length, containing a typical set of 37 genes (13 protein-coding genes, 2 rRNA genes, 22 tRNA genes) and a 346bp non-coding A+T-rich region. Phylogenetic analysis using mitochondrial genomes of 40 species showed that *A. sanguiflua* formed a well-supported monophyletic group with other Zygaenidae species.

*Amesia sanguiflua* (Lepidoptera: Zygaenidae) is a diurnal moth with colorful and diverse spots wing patterns, its larva is a predator of the plants of Proteaceae. In this study, the adults of *A. sanguiflua* were collected from Jinxiu Yao Autonomous Country (24.08°N; 110.11°E), Guangxi Zhuang Autonomous Region, China. *A. sanguiflua* was stored in ethanol and kept in the insect collection room of College of Life Sciences, Shaanxi Normal University, Xi’an, China (Voucher specimens Number: SNU-Lep-20180016). Total DNA was extracted using the TIANamp MicroDNA Kit (Tiangen Biotech, Beijing, China) according to the manufacturer’s instructions.

The conserved primers (Simon et al. 2006) had been used to amplify contiguous, overlapping fragments of the complete mitogenome sequence of *A. sanguiflua*. All PCR products were directly sequenced by PCR primers with a primer-walking strategy. These fragments were assembled into a complete mitochondrial DNA sequence using the Staden Package v1.7.0 (Staden et al. 2000). Protein-coding genes (PCGs) and rRNAs were identified based on NCBI BLAST function. Positional confirmation and prediction of secondary structures of the tRNAs were identified by tRNAscan-SE (Lowe and Eddy 1997). The base composition and codon usage were analyzed using MEGA7 (Kumar et al. 2016).

The complete mitochondrial genome of *A. sanguiflua* (GeneBank accession number MK224510) was a double-stranded, circular molecular structure with 15,203 bp in length and consisted of 13 PCGs, 2 rRNA genes, 22 tRNA genes, and a control region. All 13 PCGs used ATN as the start codon, 6 PCGS (*COI*, *COII*, *ND2*, *ND4*, *ND4L*, and *ND5*) used T or TA and the other PCGs used typical TAA as the stop codon. The control region of *A. sanguiflua* was located between *rrnS* and *trnM–trnI–trnQ* with 346 bp in length. The *12s* and *16s rRNA* genes of *A. sanguiflua* mitogenome were located between *trnL^CUN^* and the control region and was separated by *trnV*, and were 769 and 1337 bp in length, respectively. Twenty-one tRNA genes could fold into the typical cloverleaf secondary structure, *trnS^AGN^* gene formed a loop due to the lack of the DHU arms. The overall base composition of the mitogenome of *A. sanguiflua* was A 40%, T 39.8%, C 12.4%, and G 7.8%. All sequenced mitogenomes of Ditrysia have the order *trnM–trnI–trnQ* (Park et al. 2016). Another cluster of tRNA was observed in both sequenced species of Zygaenidae, *trnA–trnR–trnN–trnE–trnS–trnF* (Lavrov and Lang 2005). The concatenated nucleotide sequences of 13 PCGs were used to construct phylogenetic relationships by using Bayesian and ML methods. Phylogenetic analysis suggested that the monophyly of Zygaenidae was well supported, *A. sanguiflua*, *Rhodopsona rubiginosa* (GeneBank accession number NC025761), *Eterusia aedea* (NC038208), *Histia rhodope* (NC039447), and *Pidorus atratus* (NC037909) were clustered together into a monophyletic group Zygaenidae (Tang et al. 2014; Peng et al. 2017; Bao et al., 2019; Wang et al. 2018). Limacodidae and Zygaenidae constitute a paraphyletic group (Figure 1).

**Figure 1.**
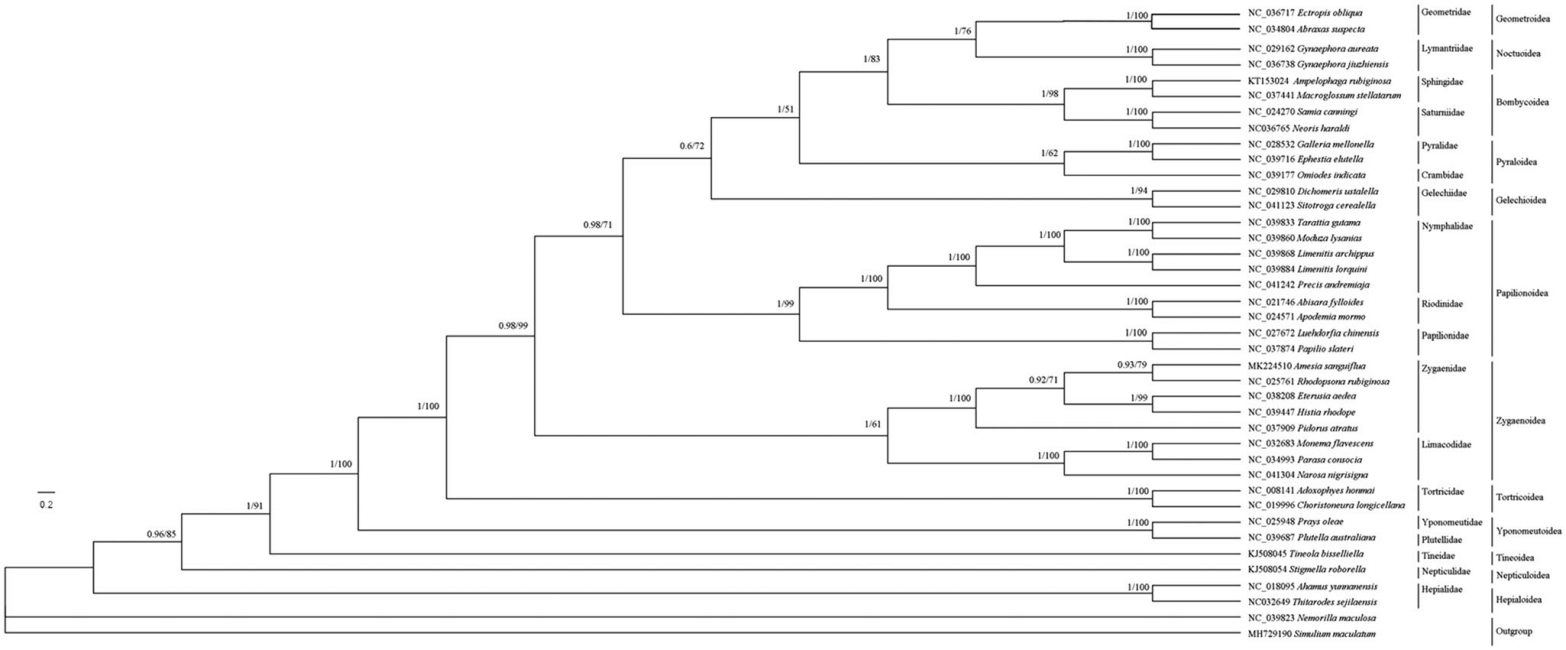
Mitogenomic phylogeny of 40 Lepidoptera species inferred from the 13 PCGs dataset based on the ML and BI analyses.

## Nucleotide sequence accession number

The complete genome sequence of *Amesia sanguiflua* has been assigned GenBank accession number MK224510.
